# Initial state perturbations as a validation method for data-driven fuzzy models of cellular networks

**DOI:** 10.1186/s12859-018-2366-0

**Published:** 2018-09-21

**Authors:** Lidija Magdevska, Miha Mraz, Nikolaj Zimic, Miha Moškon

**Affiliations:** 1 Faculty of Computer and Information Science, University of Ljubljana, Večna pot 113, Ljubljana, 1000 Slovenia; 2 Faculty of Mathematics and Physics, University of Ljubljana, Jadranska ulica 19, Ljubljana, 1000 Slovenia

**Keywords:** Fuzzy logic, Model validation, Data-driven modelling, Dynamic modelling, MAPK signalling pathway, Circadian clock

## Abstract

**Background:**

Data-driven methods that automatically learn relations between attributes from given data are a popular tool for building mathematical models in computational biology. Since measurements are prone to errors, approaches dealing with uncertain data are especially suitable for this task. Fuzzy models are one such approach, but they contain a large amount of parameters and are thus susceptible to over-fitting. Validation methods that help detect over-fitting are therefore needed to eliminate inaccurate models.

**Results:**

We propose a method to enlarge the validation datasets on which a fuzzy dynamic model of a cellular network can be tested. We apply our method to two data-driven dynamic models of the MAPK signalling pathway and two models of the mammalian circadian clock. We show that random initial state perturbations can drastically increase the mean error of predictions of an inaccurate computational model, while keeping errors of predictions of accurate models small.

**Conclusions:**

With the improvement of validation methods, fuzzy models are becoming more accurate and are thus likely to gain new applications. This field of research is promising not only because fuzzy models can cope with uncertainty, but also because their run time is short compared to conventional modelling methods that are nowadays used in systems biology.

## Background

Computational models are depictions of reality that help us understand biological systems and direct experimental work in the field of systems biology [1]. A diverse range of methods for building models is available nowadays, with data-driven approaches playing an important role in cases where a large amount of experimental data exists and where prior knowledge of the system’s structure is limited. A major advantage of these methods is that they can incorporate data directly without the need for expert knowledge to interpret the data, as their aim is to find correlations between data attributes [2, 3].

With experimental data, a certain level of measurement error appears [4]. A promising approach to dealing with this problem are Bayesian networks that allow the incorporation of qualitative data into the structure of the network, the likelihood function and the prior probability distribution of Bayes’ rules [5], with a drawback that the prior probability distribution may sometimes not be available [6]. An alternative approach is fuzzy logic.

Fuzzy logic is an extension of traditional Boolean logic. The concept of a linguistic variable provides a means of approximate characterization of phenomena which are too complex or too ill-defined to be applicable in conventional quantitative terms [7]. To build a model, for each variable its term-set, the collection of linguistic (fuzzy) values, and a membership function are defined. Additionally, a set of fuzzy terms in the form of ’IF-THEN’ rules is constructed, defining the relations between linguistic variables [8]. Fuzzy models of cellular networks have been presented in [3, 6, 9–12].

Fuzzy models contain a large amount of parameters, hence they are susceptible to over-fitting. Additionally, it is possible that simulation results on small testing datasets fit the modelled system equally well for models with different sets of parameter values and topologies. This is especially likely in case of data-driven models as algorithms that build them do not account for the biological system’s topology and may as such find a completely unsuitable solution. It is therefore important to expand the validation dataset in a way that helps us distinguish between accuracies of models with different topologies.

Computational models are typically validated on available experimental datasets and data that is collected from experiments that are performed after the establishment of the model. Models of signalling pathways often assume that the system’s response only depends on the stimulus concentration [6, 13, 14], while they ignore the initial state of the system at the time of stimulation of the pathway. On the other hand protein concentrations are known to vary between cells and inside the same cell in different time points from 15 to 30% of their mean value [15]. This suggests that perturbations of protein initial concentrations could provide a successful method for fuzzy model validation.

First we apply our validation method to two fuzzy models of the classical cascade of the mitogen-activated protein kinase – MAPK. It is the most studied pathway from the MAPK signaling cascade family and coordinates many cellular activities in eukaryotic cells, such as gene expression, mitosis, metabolism, survival, apoptosis, and differentiation [16]. In cases where this signalling pathway is damaged, diseases such as cancer, Alzheimer’s and Parkinson’s disease may occur [17].

Later we apply the method to two fuzzy models of the mammalian circadian clock – CC, a timing system that forms rhythmic changes of processes in the body, with a period close to 24 h, allowing organisms to adapt to the cyclic changes in their habitats [18]. The disruption of this clock may cause a variety of pathologies, including cardiovascular and inflammatory diseases, cancer, and depression [19–22].

Many models have been built to analyse the dynamics of both systems. These models, however, use conventional computational biology methods [23–32] that have a long execution time and cannot deal with uncertain data.

## Methods

### Training, testing and validation datasets

Training, testing and validation sets for the MAPK signalling pathway were generated from the model presented in [23]. The model is based on ordinary differential equations (ODEs) and was run in MATLAB for a time span of 30 min using the built-in ode45 function, with data being collected once per minute. Training and testing data were generated with constant initial conditions and variation of the epidermal growth factor – EGF (stimulus) concentration. All perturbations of the EGF concentration were inside the range that was experimentally tested in [23]. The validation set was generated by random perturbations of both initial conditions and EGF concentration. Training set of the mammalian CC was generated from the findings published in [32] following the recommendations of [33]. As test and validation datasets the raw data measured in liver under dark-dark conditions [32] were used.

### Data-driven fuzzy models

In this article, two algorithms for building fuzzy models are used. Both algorithms use Zadeh-Mamdani fuzzy rules [34] that are of the form

1 $$ \text{IF}\ x \ \text{is}\ \tilde{A}\ \text{THEN}\ y\ \text{is}\ \tilde{B},  $$

where (*x* is $\tilde {A}$) and (*y* is $\tilde {B}$) are two fuzzy terms. The input variable *x* belongs to the fuzzy set $\tilde {A}$ with the membership function value $\mu _{\tilde {A}}(x)$, and the output variable *y* belongs to the fuzzy set $\tilde {B}$ with the membership function value $\mu _{\tilde {B}}(y)$. A general form of this rule that allows us to use an arbitrary number of input and output variables is

2 $$\begin{array}{*{20}l} \text{IF}\ x_{1}\ \text{is}\ \tilde{A}_{1}\ \text{AND}\ x_{2}\ \text{is}\ \tilde{A}_{2}\ \text{AND}\ \hdots\ \text{AND}\ x_{k_{1}}\ \text{is}\ \tilde{A}_{k_{1}} \\ \text{THEN}\ y_{1}\ \text{is}\ \tilde{B}_{1}\ \text{AND}\ y_{2}\ \text{is}\ \tilde{B}_{2}\ \text{AND}\ \hdots\ \text{AND}\ y_{k_{2}}\ \text{is}\ \tilde{B}_{k_{2}}. \end{array} $$

For input and output variables we assume a Gaussian membership function that is defined with a mean value *c* and standard deviation *σ*, and is calculated from the expression

3 $$ \mu_{\tilde{A}}(x) = e^{- \frac{(x-c)^{2}}{2\sigma^{2}}}.  $$

For defuzzification of output variables, the center of gravity (COG) method [35] is used. The crisp value *R*^′^ of a result of processing *R* that is described with a continuous membership function $\mu _{\tilde {R}}(y)$ equals

4 $$ R' = \frac{\int_{0}^{\infty}{y \mu_{\tilde{R}}(y)dy}}{\int_{0}^{\infty}{\mu_{\tilde{R}}(y)dy}}.  $$

Additionally, we assume that the next state of the system only depends on the previous state and the value of the stimulus.

#### Fuzzy c-means clustering algorithm (FCM)

The fuzzy c-means clustering algorithm (FCM) [36] is a basic fuzzy algorithm for clustering that searches for a fuzzy partition *U*=[*u*_*ik*_] of data collection by minimising the generalised least squares functional

5 $$ J_{m}(X,U,v) = \sum\limits_{k=1}^{N}\sum\limits_{i=1}^{c} u_{ik}^{m} d^{2}(x_{k},v_{i}),  $$

where $X= \{x_{1}, x_{2}, \hdots, x_{N}\} \subset \mathbb {R}^{n}$ is a set of data, *c* the number of clusters in the set *X* (2≤*c*<*N*), *m*≥1 the degree of fuzzification to remove noise from data, *d* a distance function, *U* the fuzzy partition of set *X*, and *v*=[*v*_*i*_] the vector of cluster centres. The minimisation is run iteratively under the following conditions:

6 $$\begin{array}{*{20}l} &0 \leq u_{ik} \leq 1; \; 1 \leq i \leq c, 1 \leq k \leq N, \end{array} $$

7 $$\begin{array}{*{20}l} &0 < \sum\limits_{k=1}^{N} u_{ik} \leq n; \; 1 \leq i \leq c, \end{array} $$

8 $$\begin{array}{*{20}l} &\sum\limits_{i=1}^{c} u_{ik} = 1; \; 1 \leq k \leq N. \end{array} $$

After each iteration, centres *v*_*i*_ and membership degrees *u*_*ik*_ are updated using the following procedure:

9 $$\begin{array}{*{20}l} v_{i} &= \frac{{\sum}_{k=1}^{N} u_{ik}^{m} x_{k}}{{\sum}_{k=1}^{N} u_{ik}^{m}}; \; 1 \leq i \leq c, \end{array} $$

10 $$\begin{array}{*{20}l} u_{ik} &= \frac{1}{{\sum}_{j=1}^{c} \left(\frac{d(x_{k},v_{i})}{d(x_{k},v_{j})}\right)^{\frac{2}{m-1}}}; \; 1 \leq k \leq N, 1 \leq i \leq c. \end{array} $$

For a fuzzy model with *n* input and *m* output variables, its learning with FCM uses (*n*+*m*)-dimensional vectors as data, where each vector contains known values of input and expected values of output variables at given learning inputs. These data are then clustered in *c* groups with every group representing one fuzzy rule. Membership functions of fuzzy variables are determined from the groups’ centres.

In the case of a cellular network model the input variables are concentrations of chemical species, while the output variables are the changes in concentrations of chemical species in two consecutive measurements. The change of concentration of the stimulus is ignored, as we assume that it is constant throughout the whole simulation time span. Since the training and testing datasets contain absolute concentration values, the learning method determines the changes, while the final model computes absolute values from input values and fuzzy model outputs.

This learning method is performed using the MATLAB function genfis3. Since its results are non-deterministic, the method is run 10 times and the model with the smallest error on the training set is selected for further observations.

#### Multi-atribute fuzzy time series method

Fuzzy time series is a prediction model that allows modelling dynamic processes in which linguistic values are observed. The model assumes that an observation in a time point is the result of observations from the past [37]. One of the procedures to build a fuzzy time series is the multi-atribute fuzzy time series method [38], later denoted as MAFTS. It consists of four steps:

1. The clustering of time series *S*(*t*) into *c* clusters using FCM to identify patterns,
2. The ranking of each cluster and fuzzification of time series *S*(*t*) to a fuzzy time series *F*(*t*),
3. The determination of fuzzy rules,
4. The prediction of new data and defuzzification of results.

Data used for clustering is a set of concentrations of chemical species. The data of each chemical species is clustered separately to determine membership functions of the corresponding variable. Mean values of the Gaussian membership functions are determined as cluster centres obtained by FCM, while standard deviations are set to a constant percentage (3.5% in case of the MAPK signalling pathway and 0.8% in case of the CC) of the length of the interval on which a fuzzy variable is defined, in order to reduce the number of parameters that have to be learnt. Since membership functions for each protein are determined separately, linguistic names can be given to linguistic values. Each fuzzy variable gets either 3 or 5 fuzzy values denoted *low*, *medium*, and *high* (with 5 fuzzy values also *very low*, and *very high*), so that their mean values correspond to the linguistic meaning of the linguistic values. The number of fuzzy values per variable was set as in [6, 10], but could be extended in case of inaccuracy of the built model or reduced in case of over-fitting. The domain of a fuzzy variable is defined as a closed interval from 0 to the maximum value achieved by the variable on the training data.

Data points are fuzzified so that the fuzzy value with the maximal membership function value is chosen for each fuzzy variable. For each pair of consecutive data points, one fuzzy rule is determined. Fuzzy values of the fuzzy variables at the earlier time point are included in the IF part of the rule, and the fuzzy values at the later time point in the THEN part of the rule. Input and output variables of the fuzzy model are hence concentrations of chemical species. The stimulus concentration is not predicted as we assume that it is constant through the whole simulation time span.

The MATLAB function fcm is used to cluster protein concentrations. Since its results are non-deterministic and it sometimes returns results of numeric type NaN, learning is repeated until a valid numeric result for cluster centres is obtained.

### Model evaluation metric

Model accuracy is evaluated using a mean absolute error (MAE)

11 $$ \text{MAE} = \frac{{\sum}_{i=1}^{n} \text{abs}(\epsilon_{i})}{n},  $$

and a root mean square error (RMSE)

12 $$ \text{RMSE} = \sqrt{\frac{{\sum}_{i=1}^{n} \epsilon_{i}^{2}}{n}},  $$

where *n* denotes the number of test instances and *ε*_*i*_ the prediction error of the *i*-th test instance [39]. The prediction error is measured as the average normalized difference between the true values and the predicted values of a component (variable) within a test instance. Each component was normalized by the maximal value of its domain.

## Results and discussion

In order to gather validation data for dynamic models, experimental data needs to be sampled in a series of time-points after perturbations of experimental conditions. An appropriate design of time-series experiments is difficult and may contain redundant information leading to the inefficient use of experimental resources [40]. An alternative approach for model validation is therefore a comparison with existing models that allows us to sample validation data of arbitrary size. This is especially useful when accurate models exit, but are too slow to be effectively incorporated in experimental work.

### Fuzzy model of the MAPK signalling pathway

We generated two data-driven fuzzy models of the MAPK signalling pathway from the same training dataset. The first model was generated using FCM with 20 clusters and the second model with MAFTS with 5 fuzzy values per variable. Both models simulate the dynamics of the MAPK signalling pathway by iterative runs of the inference system. Given an initial condition and EGF concentration models returns a time series of 30 consecutive states of the system.

We are searching for a model that describes the dynamics of a signalling pathway. In contrast to some prediction models, where, given a state, the model has to produce an accurate prediction of the next state (i.e. the state in the next time point), later called *next state prediction*, we attempt to find a model that given an initial condition and a stimulus concentration, predicts an accurate series of consecutive states. We call the later a *whole time series prediction*.

MAE and RMSE were hence calculated on two testing sets and two validation sets. One of the sets used the predictions of the next state from a given state, while the other predicted a series of states from a given initial state.

The errors of the generated fuzzy models were of similar size for the testing sets that included the results of a whole time series, while the next state prediction was better using the model generated with FCM (Table 1). At this stage of validation, we could thus assume that the model generated with FCM is either more accurate than the model generated with MAFTS or that they are both approximately as accurate.

**Table 1:** Test sets errors

	FCM model	MAFTS model
MAE (next state)	0.07	0.14
MAE (whole series)	0.76	0.24
RMSE (next state)	0.02	0.10
RMSE (whole series)	0.47	0.15

MAE and RMSE measured on models generated with FCM and MAFTS with respect to the testing sets where either the next state or a whole time series is predicted

We then generated validation data with initial state perturbations to validate our assumption. Validation data were generated with two distinct approaches. In the first case only the initial state was randomly selected so that it belonged to the domain on which the models are defined, while the EGF concentration was randomly taken from the set of EGF concentrations that occur in training data. In the second case both the initial state and stimulus concentration were randomly selected from the domain. MAE and RMSE were measured as before.

We found out that in both cases errors of the model generated with FCM increased notably compared to the testing data (Tables 2 and 3), while the errors of the model generated with MAFTS increased only slightly. The main reason for the increase of the whole series prediction error of the model generated with FCM is that the model estimates the difference in concentration and not the concentration itself, allowing the concentration prediction to increase above the maximum value of the domain. Once the input variables of the FCM model are outside the domain, the results are unlikely to be in the domain, leading to large errors. Such errors are likely to occur whenever replacing ODE models with fuzzy models with an aim to speed them up.

**Table 2:** Errors on validation sets with initial state perturbations

	FCM model	MAFTS model
MAE (next state)	0.20∗10^3^	0.15
MAE (whole series)	1.41∗10^3^	0.24
RMSE (next state)	3.28∗10^3^	0.22
RMSE (whole series)	8.67∗10^3^	0.31

MAE and RMSE measured on models generated with FCM and MAFTS with respect to the validation sets with initial state perturbations where either the next state or a whole time series is predicted

**Table 3:** Errors on validation sets with initial state and stimulus concentration perturbations

	FCM model	MAFTS model
MAE (next state)	0.29∗10^3^	0.16
MAE (whole series)	2.02∗10^3^	0.25
RMSE (next state)	4.35∗10^3^	0.23
RMSE (whole series)	11.5∗10^3^	0.31

MAE and RMSE measured on models generated with FCM and MAFTS with respect to the validation sets with initial state and stimulus concentration perturbations where either the next state or a whole time series is predicted

Our results show that the model generated with MAFTS is much more accurate than the model generated with FCM, although we were unable to form this conclusion from the testing datasets generated by exclusively EGF concentration perturbations. These findings suggest that perturbations of initial conditions can simplify the process of model validation as even a small dataset can sometimes eliminate an inaccurate fuzzy model.

### Fuzzy models of the mammalian circadian clock

The observations of the models of the MAPK signalling pathway might suggest that sensitivity to perturbations is a feature of FCM models. For this reason we generated two data-driven fuzzy models of the mammalian circadian clock from the same training dataset using MAFTS. In the first case we used 3 fuzzy values per variable, and in the second case we used 5 fuzzy values per variable. Both models again simulate the dynamics of the network by iterative runs of the inference system.

Korenčič et al. [32] suggests that the effect of transcription factors on gene expression at a given time point can be modelled as an effect of gene expression levels at earlier time points. This delay corresponds to the time needed for post-transcriptional modifications and differs between genes. In order to integrate this approach to MAFTS, the previous state was defined as a set of gene expression levels before delay time points. The initial condition in this case is therefore a series of four states, as the largest delay observed in [32] corresponds to four hours. In each model a series of 24 states corresponds to the 24 h day cycle. As with the previous case study we attempt to find a model that, given an initial condition, predicts an accurate series of consecutive states, however, in this case it is more important that the system keeps oscillating than to obtain low MAE or RMSE. Without any initial state perturbations both models produced oscillations with a 24 h period.

**Fig. 1:**
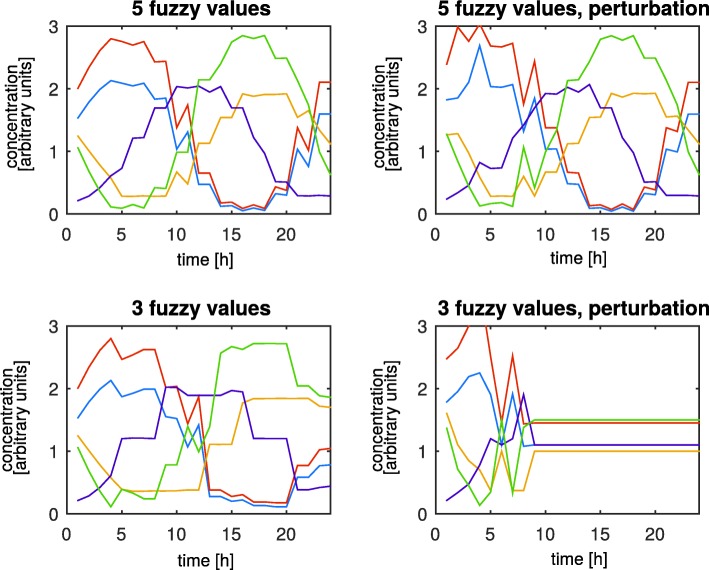
Comparison of fuzzy models of the circadian clock. Simulation results of both fuzzy models. After initial state perturbations the model with 5 fuzzy values per variable keeps oscillating, while the model with only 3 fuzzy values stops. Without initial state perturbations both models showed oscillations with a period of approximately 24 h

Perturbations of initial conditions were up to 1% of their value, which is less than the differences between measurements in different mice at the same time point in [32], meaning that they should not affect the dynamics of the system. As Fig. 1 shows the model with 5 fuzzy values per variable keeps oscillating, while the model with only 3 fuzzy values stops oscillating after 10 h of simulation.

While in this case the inaccuracy is not a consequence of over-fitting, we show that initial state perturbations can also help as a testing method to determine the minimal number of fuzzy values needed to accurately describe the dynamics of a cellular network.

### Discussion

The size of available datasets limits many validation methods not only due to the complexity of the experimental work, but also due to the long runtime of simulations of large ODE and partial differential equations (PDE) models that are still the most popular approach for the depiction of signalling pathways and gene regulatory networks. This also holds true for the reference ODE model used in this study, but we were still able to generate a validation dataset of sufficient size to disprove the fuzzy model generated with FCM.

This limitation should, however, not prevent one from using the proposed method, as simulations of fuzzy models are much faster than the corresponding ODE reference models and several fuzzy models can be validated using the same validation datasets. Additionally, our method can be extended to cases where appropriate experimental data or any type of an accurate quantitative model of the observed biological system is available.

## Conclusions

Validation of computational models of biological systems is often problematic, as only small experimental datasets are available for comparison. In this paper we provided a description of an approach that helps in eliminating inaccurate fuzzy data-driven models through initial state perturbations of a dynamic system. We demonstrated the method’s applicability by comparing two data-driven fuzzy models of the MAPK signalling cascade and two data-driven fuzzy models of the mammalian CC, where we successfully detected an over-fitted model. With the improvement of validation methods fuzzy models are not only becoming more accurate, but are also becoming a more promising alternative to conventional modelling methods as they can cope with uncertain data and can predict outputs quickly. The presented method can be also extended to the validation of fuzzy dynamic models of a diverse spectrum of biological systems, providing an opportunity for new applications of fuzzy logic to systems biology. The latter can gain importance through data-driven models built directly from experimental data or as a way to speed up existing models that are accurate but too slow for frequent usage.

## Abbreviations

CC: Circadian clock
EGF: Epidermal growth factor
FCM: Fuzzy c-means clustering algorithm
MAE: Mean absolute error
MAFTS: Multi-atribute fuzzy time series method
MAPK: Mitogen-activated protein kinase
ODE: Ordinary differential equations
RMSE: Root mean square error
